# Allergy-Related Disorders in the Construction Industry

**DOI:** 10.5402/2013/864679

**Published:** 2013-12-05

**Authors:** Mauro Carino, Paolo Romita, Caterina Foti

**Affiliations:** ^1^Occupational Health Unit, National Health Service, Lungomare Starita 6, 70123 Bari, Italy; ^2^Department of Biomedical Science and Human Oncology, Unit of Dermatology, University of Bari, 70124 Bari, Italy

## Abstract

Working conditions in the construction industry have improved in many industrialized countries, but heavy physical work with recurrent exposure to chemical agents, dust, and climatic influences still represents considerable risk for construction workers and may affect their health. The aim of this review is to analyze available data of the literature on allergy-related respiratory and skin disorders with emphasis on a preventive appraisal in order to produce statements and recommendations based on research evidence. The most common agents involved in the construction industry as a cause of occupational asthma (OA) in industrialized countries are isocyanates, wood dust, resins, glues, cobalt, and chromium. Allergic contact dermatitis (ACD) is an immunologic cell-mediated response to a sensitizing agent and the most common sensitizing agents associated with construction workers are epoxy resins, thiurams and thiazoles, and chromates. Medical surveillance must consider individual risk factors such as differences in individual susceptibility and sensitization to agents at workplace. Once work-related disorder is confirmed, adequate fitness for work should be assessed for the worker impaired by health condition. A reliable diagnosis of an index case is a sentinel event that may reveal risks for workers with similar exposure, leading to a revised risk assessment at the workplace that should reduce the risk and prevent further cases.

## 1. Introduction

Construction industry plays a major role in the economic growth of a nation and construction workers are at increased risk of work-related disorders worldwide [1, 2]. Allergic diseases represent a major health problem in most developed countries and are associated with serious adverse health and socioeconomic outcomes [3, 4]. Working conditions in the construction industry have improved in many industrialized countries during past decades, but heavy physical work with recurrent exposure to chemical agents, dust, and climatic influences still represents considerable risk for construction workers and may affect their health [5]. Among workers with similar occupational exposures, diagnosis of allergy-related disorders in the construction industry offers unique opportunities for prevention. The aim of this review is to analyze available data of the literature on these diseases with emphasis on preventive aspects in order to produce statements and recommendations based on research evidence.

## 2. Respiratory Disorders

Exposures in the workplace continue to contribute to asthma morbidity among adults and is a cause of disability and economic consequences for both the worker and the society [6, 7]. Asthma at worksite often goes unrecognized [8] and a correct and early diagnosis is important to limit consequences of the disease [9]. Several hundred of occupational agents have been identified as causing work-related asthma, mainly allergens but also irritants such as ammonia, chlorine, sulfur dioxide and substances with unknown pathogenic mechanism [10]. Work-related asthma is currently one of the most common occupational respiratory diseases in many industrialized countries and 15–25% of adult asthmatic patients are estimated to have asthma attributable to occupational factors [11, 12]. Main new categories of responsible occupational agents reported in the last 10 years have been recently reviewed [13]. The possibility that nonoccupational physicians face this disorder in daily practice is high and they may play a crucial role in identifying suspected cases.

Work-related asthma includes two major disorders: (1) occupational asthma (OA) (caused by well-established causative agents at workplace) (2) work-exacerbated asthma (triggered by various work-related factors such as aeroallergens, irritants, or exercise in workers who are known to have preexisting or concurrent asthma occurring at workplace). Two types of OA are distinguished (a) allergic: it is the most common type with more than 90% of the cases [14] and it appears after a latency period necessary for the worker to be sensitized to the causal agent (mostly high and some low molecular weight agents with IgE-mediate mechanism) (b) nonallergic: irritant-induced OA such as the Reactive Airways Dysfunction Syndrome (RADS) which occurs as an acute onset of asthma after high level single exposure to an irritating gas, smoke, or vapor [15]. A few cases of irritant-induced OA with a not sudden onset of asthma that follows multiple exposures to high levels of irritants are reported [16].

Medical histories taken by experts have high sensitivity, but lower specificity [17]. A specialist should identify specific exposures with a systematic occupational history, examination of safety data sheets, industrial hygiene measurements and internal reports. The failure to find a sensitizer on these sources should not preclude the diagnosis of OA, as many sensitizers are not regularly listed, particularly those in low concentration, those that are only present in definite circumstances, such as when heated and those given nonspecific titles such as resin and fragrance. Spirometry is required in all suspected patients. Measurement of nonspecific airway responsiveness such as challenge with methacholine is part of the diagnostic assessment in patients with no significant baseline obstruction. The skin prick test performed according to international guidelines is the main step of allergological testing and is generally regarded as sensitive, but less specific [18]. Serum specific IgE tests are often less sensitive, but possibly more specific than skin tests. Specific inhalation challenge tests are commonly regarded as the golden standard method for diagnosing work-related asthma induced by sensitizers, but in practice the test is scarcely available and not internationally standardized. The role of novel methods for monitoring the inflammatory activity in the airways such as the measurement of exhaled nitric oxide or exhaled breath condensate is promising; however, at present their definite place in practical diagnosis is not clear.

The potential health benefit of a correct and early diagnosis of work-related asthma is substantial for the individual, industry, and society. However, the possibility of an asthma related to the worksite is not frequently considered and adequate measures to secure an etiologic diagnosis are not performed. An integrated approach is recommended and an organized collaboration between occupational, primary care, and specialized physicians is required. Once work-related asthma is confirmed, a revised risk assessment at the workplace is needed to prevent further cases. Consensus statements and guidelines for investigation and management of work-related asthma, primary prevention concerning exposure reduction, skin exposure, and respiratory protection have been recently published by European Scientific Societies in the field of allergy and clinical immunology and occupational and respiratory medicine [19–22].

### 2.1. Identification of Causes at Worksite: Agents and Tasks

The most common agents involved in the construction industry as a cause of OA in different industrialized countries such as Canada, Finland, France, Great Britain, Italy, and United States are isocyanates, wood dust, resins, glues, cobalt and chromium. The prevalence of each of them on the total number of cases in each geographical area varies in the different periods considered between 0.5 and 25.2% [23]. OA due to sensitization or OA with latency period is certainly the most common diagnosis. The latency of onset of symptoms after exposure to the substance varies from a few weeks to many months, even in the case of low exposures. In contrast, OA without latency period usually follows concentrated exposure to high doses of irritants to the respiratory tract. It should also be mentioned that preexisting asthma symptoms can be aggravated at building worksite.

A literature review carried out in order to calculate the population attributable risk (PAR) as risk of asthma in the general population attributable to work exposure showed particularly high values for the construction industry [24], data confirmed also by other surveys conducted on a large scale in the United States [25]. The best evidence to date has been provided by a twenty-year followup Finnish study that has defined the age-adjusted relative risks (RR) of asthma for 24 tasks of an adult male population in the construction industry [26]. The risk was increased in nearly all construction occupations studied, but it was the highest among welders and flame cutters (RR 2.34), asphalt roofing workers (RR 2.04), plumbers (RR 1.90), brick layers, and tile setters (RR 1.83). Only 45 (2%) of the cases of asthma among construction workers had been recognized as OA. Construction industry workers have an increased risk of adult-onset persistent asthma and cases of OA caused by well-established causative agents have only a minor contribution to this overall asthma excess. There are many chemicals used in the construction industry, with a rapid and continuous introduction into the market of new products including insulating materials, glues, and additives used in cements and plasters. There are, for instance case reports of acute respiratory illness following exposure to certain formulation of stain-repellent waterproofing resins containing acrylate fluoropolymers [27]. A study in a European small size industrialized city considering the population aged between 20 and 59 years showed a significant association (OR 3.38) between symptoms of chronic bronchitis and working in the building industry [28]. An excess of cases of bronchial obstruction (defined as FEV1/FVC < 75% and FEV1 < 80% predicted) attributable to occupational exposure in the construction sector was found during a prevalence survey in the US general population [29] and a case-control study conducted in Italy confirmed these data showing a higher risk of bronchial obstruction (OR 3.13) for the occupational tasks in the construction industry [30].

A dataset from a voluntary surveillance scheme for reporting cases of medically diagnosed occupational disease has been used to describe the overall incidence of work-related ill health in the United Kingdom construction. Suspected causal agents for asthma reported by respiratory and occupational physicians are shown in Table 1. The most common causal exposures in metal and electrical tradesmen were isocyanates (from paint spraying) and metalworking fluids. For building and construction tradesmen, these were wood and wood dust.

### 2.2. Medical Surveillance, Fitness for Work, and Compensation Issues

Medical surveillance must consider individual risk factors such as differences in individual susceptibility and sensitization to agents at workplace [32, 33]. According to the different working contest, it could be reasonable when assessing fitness for work for subjects showing nonspecific bronchial hyperresponsiveness and allergic rhinitis to completely avoid exposure because of their higher risk of developing asthma [34]. However the positive predictive values of available susceptibility markers are too low for screening out potentially susceptible individuals [35]. This is particularly true in the case of atopy and smoking, which are highly prevalent in the general population. Excluding atopic individuals from jobs with exposure to high molecular weight allergens would drastically restrict potential new recruitments [36, 37]. Medical surveillance aims to identify susceptible workers and comprehensive medical surveillance programs should be performed especially in high-risk groups such as subjects with rhinitis, nonspecific bronchial hyperresponsiveness, sensitization to common allergen such as pollens, mites, and molds, subjects exposed to high molecular weight allergens or high concentrations of irritant chemicals. It should include preplacement and periodic administration of a questionnaire, skin-prick tests, or measurement of specific serum IgE antibodies when an exposure to a respiratory allergen is established. The frequent short latency period for the development of OA implicates that surveillance programs for individuals at risk need to begin during vocational training [38, 39]. Early referral of symptomatic and/or sensitized workers for specialized medical assessment is mandatory. These worker-related investigations need accompanying exposure assessment at the workplace and appropriate interventions targeted toward workers and worksite.

In clinical practice the optimal management of OA remains uncertain. A systematic literature search was conducted to identify original studies addressing different options such as persistence of exposure, reduction of exposure, complete avoidance of exposure, use of personal protective equipment pharmacological treatment. The conclusions from this review after analysis of fifty-two studies are limited because of questionable methods used in some published studies [40]. Critical investigation of available evidence indicates: (a) persistent exposure to the causal agent is more likely to result in asthma worsening than complete avoidance, (b) avoidance of exposure leads to recovery of asthma in less than one-third of affected workers, (c) reduction of exposure seems to be less beneficial than complete avoidance of exposure, (d) personal respiratory equipment does not provide whole protection, and (e) there is insufficient evidence to determine whether pharmacological treatment can alter the course of asthma in subjects who remain exposed. Once work-related disorder is confirmed, adequate fitness for work should be assessed for the worker impaired by health condition, since persistence of exposure to an agent causing work-related asthma leads to a worsening of the disease and even life-threatening consequences [41]. The severity of these disorders should be assessed according to acknowledged grading patterns. A growing consensus considers that surveillance and compensation systems should be directed to accommodating workers in unexposed jobs possibly within the same company and to offering specific rehabilitation programs if required. The policies governing compensation of respiratory disorders vary widely from one country to another. These differences are caused by administrative regulations, different criteria for definition and determination of causation (e.g., burden of proof charged to the worker for causal agents not present in the official list of national regulation), and evaluation of disability level. The criteria used for determining eligibility for compensation are not uniform and according to regulations of a particular country may cover different aspects of physiological impairment, work disability, healthcare costs, loss of income, and professional retraining. Available data for OA and work-aggravated asthma indicate that financial compensations do not adequately counterbalance the socioeconomic consequences of the diseases [42, 43].

## 3. Skin Disorders

Construction workers perform several duties that may include mixing and handling irritant and sensitizing materials such as concrete, cement, and asphalt. They often get in contact with these latter, in particular when performing small jobs, despite the widely used mechanized methods of construction. Moreover, they work under different weather conditions including hot, wet, and cold ones. These evidences may explain why occupational dermatitis is widespread among this particular category of workers [44–46]. Cement is steadily used in the construction sector and unfortunately workers often do not realize that it is a chemical. What is more, cement has constituents that may cause both irritant (from alkaline ingredients) and allergic contact dermatitis (from ingredients such as chromium): contact dermatitis is, as a matter of fact, the most frequently reported health problems among construction workers.

Irritant contact dermatitis (ICD) is a nonimmunologic response to a skin irritant. Severe irritants materials (strong acids or alkalis and heavy metals) cause symptoms as immediate pain and burning, followed by the quick onset of red blisters, ulcers, erosion, and necrosis. Weak irritants (solvents, synthetic oils, and sunlight) cause itching erythematous-edematous-vesicular lesions that may become chronic, for cumulative reaction over time, with lichenified scaling, cracking, fissures, and a less red erythema.

**Figure 1:**
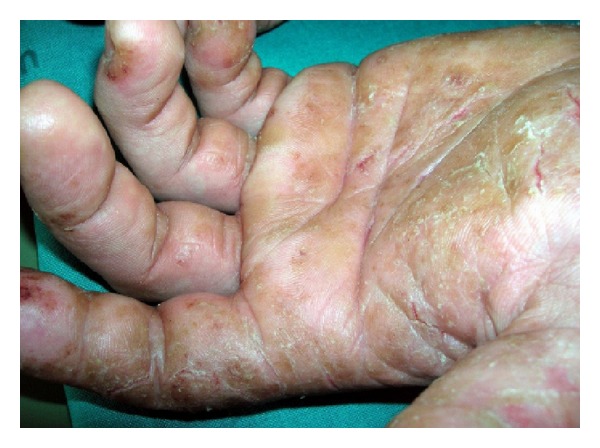
Allergic contact dermatitis with chromates in a construction worker due to cement handling.

Allergic contact dermatitis (ACD) is an immunologic cell-mediated response to a sensitizing agent. The most common sensitizing agents associated with construction workers are epoxy resins, thiurams and thiazoles, and chromates (Table 2). Epoxy resin is a plastic strong sensitizing agent widely used as electrical insulation, coatings, and adhesives [47]; thiurams and thiazoles are often present as additives in the rubber gloves used by construction workers; chromates are used as ingredients in the manufacture of products such as cement, paints, anticorrosives, and leather. Most exposure is via the workplace and ACD to chrome is usually due to dichromates found in cement [48]. In particular, ACD is the highest in incidence amongst workers handling wet cement (Figure 1).

It is recommended that construction workers should be provided personal protective equipment (PPE) and occupational health services. The regular use and maintenance of PPE as gloves, clothing, and goggles are very effective means to prevent occupational skin diseases. Moreover, many dermatitis can be prevented by improved workers and workplace cleanliness. Good personal hygiene should be emphasized and workers should be advised about proper handwashing agents.

## 4. Registration, Evaluation, Authorization, and Restriction of Chemicals (REACH): The New European Union Regulation

Attempts to identify the probability of an agent to act as respiratory or skin sensitizer preceding the extensive commercial use have been mostly performed using animal models [49]. The hazard evaluation of low molecular weight agents always includes an assessment of their dermal sensitization potential. In most cases this is now performed using an animal-based local lymph node assay, investigating the capacity of topically applied chemical agents to induce proliferative lymphocyte responses in draining nodes [50]. Chemicals that are positive to a test involving dermal application should be considered as sensitizers regardless of the way of contact with the organism, including inhalation [51]. It has not yet been assessed whether chemicals that are negative in skin sensitization tests are unlikely to cause respiratory sensitization. A different approach involving structure-activity analysis is used for low molecular weight agents; acid anhydride, amine, isocyanate and carbonyl containing nitrogen or oxygen functional groups were associated with an occupational asthma hazard, particularly when the functional group was present twice or more in the same molecule [52].

The main goal of the new European Union legislation REACH: Registration, Evaluation, Authorization, and Restriction of Chemicals is to promote sustainable industrial development and to reduce health risks associated with use of chemicals. The REACH regulation requires that only registered substances may be manufactured or imported into the EU. It is designed to encourage the substitution of those chemicals and processes with a negative impact on health and environment. Manufacturers and Importers of a given substance must submit a registration dossier to the European Chemical Agency (ECHA) and although REACH is not directly aimed at the working environment but more focused on the consumer, it will have an impact because it prescribes under which circumstances workers may handle chemicals. The exposure circumstances for different processes and the health risks for exposed workers remain to be established. Several large groups of sensitizing agents are partially or totally exempted from REACH registration such as enzymes used in food because they are considered agents required for the process but not present in the final product. Latex, wheat flour, other natural substances, and unpackaged products are also excluded. This recent specific European Union legislation also does not cover agents such as allergens from animals or microorganisms or combustion products which have no producers they can be attributed to. Therefore the REACH regulation does not include several important allergens and irritants with an acknowledged public health impact. Even though standard setting for asthma inducing agents does have major limitations in applicability to workers and exposure, a Dutch study supports that scientifically based exposure standards for respiratory sensitizers may be derived [53].

## 5. Conclusions

The essential message of this review is that the management of allergy-related disorders in the construction industry can be considerably optimized based on the present knowledge of causes, risk factors, pathogenesis mechanisms, and effective interventions at workplace. In order to reach this goal an intensification of primary preventive measures and improvement of case management is greatly required. There is now a substantial body of evidence supporting the implementation of comprehensive medical surveillance programs for workers at risk, referring suspected workers to a clinician who must be aware of the potential occupational etiologies in order to consider them when confirming or excluding an occupational causation. The prognoses and financial burden can be improved within a framework of secondary prevention by early diagnosis within a short time window of opportunity after the onset of symptoms. A reliable diagnosis of an index case will be a sentinel event that may reveal risks for workers with similar exposure, leading to a revised risk assessment at the workplace that should reduce the risk and prevent further cases. Once a work-related disorder is confirmed, adequate fitness for work should be assessed for the worker impaired by health condition. Enhancing surveillance through an adequate optimization of primary, secondary, and tertiary preventive interventions in the construction work will allow health professionals, government institutions, employers, and worker representatives to target more effective intervention and prevention efforts to reduce the burden of allergy-related disorders.

## Figures and Tables

**Table 1:** Distribution of suspected causal agents for asthma in skilled tradesmen reported to UK Health and Occupation Reporting Network by respiratory specialists and occupational physicians from 2002 to 2008 (Source: Stocks et al., 2011) [31].

Suspected causal agents for asthma	Skilled metal and electrical tradesmen		Skilled construction and building tradesmen
	Chest physicians n = 230	Occup. physicians n = 24	Chest physicians *n* = 26
	Agents n = 240, *n* (%)	Agents n = 32, *n* (%)	Agents n = 26, *n* (%)
Metalworking fluids/coolants	59 (25)	1 (3)	0
Cobalt	8 (3)	0	0
Zinc	12 (5)	1 (3)	0
Chrome and its compounds	22 (9)	0	0
Other metals	11 (5)	0	0
Welding fumes	24 (10)	2 (6)	1 (4)
Wood and wood dust	0	0	11 (42)
Oils/greases	1 (1)	3 (9)	1 (4)
Solvents/fuel oil	5 (2)	7 (22)	0
Paints and dyes	9 (4)	3 (9)	5 (19)
Isocyanates	60 (25)	13 (41)	4 (15)
Formaldehyde	3 (1)	0	0
Other fumes and gases	7 (3)	0	0
Others	19 (8)	2 (6)	4 (15)

**Table 2:** Common causal agents of allergic contact dermatitis (ACD) in construction workers: clinical appearance and prevention.

Causal agents	Clinical features	Prevention
Epoxy resins	Erythematous-vesicular or bullous lesions vesicles, unilateral, asymmetric, involving dorsum of hands, fingers, and feet	Use of less sensitizing resin, no eating in work area. PPE: gloves and aprons washing on contact.
Chromates	Xerotic and lichenified eczema or nummular eczema involving hands that can become widespread and persistent	Change hexavalent for less sensitizing trivalent chromium; use premixed cement; periodic examination of workers' skin. PPE: gloves and work shoes; wash on contact.
Thiurams and thiazoles	Eczematous eruptions localized on hands and wrist (gloves area),	PPE: gloves that do not contain such substances (e.g., nitrile gloves)
